# Adaptive laboratory evolution and proteomic insight on collateral sensitivity for meropenem in colistin-resistant *Klebsiella pneumoniae*

**DOI:** 10.3389/fmicb.2026.1960299

**Published:** 2026-10-09

**Authors:** Sevaram Singh, Lovnish Thakur, Sapna, Shailesh Kumar, Manoj Kumar, Sameer Maitry, Shailendra Mani, Niraj Kumar

**Affiliations:** 1 BRIC-Translational Health Science and Technology Institute, NCR Biotech Science Cluster, 3rd Milestone, Faridabad–Gurugram Expressway, Faridabad, India; 2 Jawaharlal Nehru University, New Delhi, India

**Keywords:** antibiotics resistance/susceptibility, colistin, collateral sensitivity, *Klebsiella pneumoniae*, meropenem, proteomics, sub-MIC adaptation

## Abstract

**Background:**

Antimicrobial resistance (AMR) is a critical global challenge, with an estimated ≥10 million deaths per year by 2050. *Klebsiella pneumoniae* (*K. pneumoniae*), a critical priority bacterial pathogen, is rapidly developing resistance even against next-generation (meropenem) and last-resort (colistin) antibiotics, whereas the pipeline for discovery and development of new antibiotic(s) for treatment of antibiotic-resistant *K. pneumoniae* infections is a lengthy, resource-intensive, and costly process and hence demands exploring alternative and effective treatment strategies. Capitalizing on the simultaneous emergence of collateral sensitivity (CS) to resistant antibiotics can therefore be a rapid and effective strategy to fill the gap.

**Methods:**

Adaptive laboratory evolution was enforced in *K. pneumoniae* (American Type Culture Collection [ATCC] and clinical strains) by exposing it to sequentially increasing sub-lethal dosages (0.25, 0.5, and 1xMIC) of colistin and meropenem, and a panel of pathogens representing early (0.25xMIC), mid (0.5xMIC), and late (1xMIC) stages of emergence of AMR was developed. Similarly, colistin (1xMIC)-resistant *K. pneumoniae* cells were also adapted to develop resistance against meropenem. Matrix-Assisted Laser Desorption/Ionization Time-of-Flight (MALDI-TOF) for pathogen identification and VITEK-2 for antibiotic susceptibility testing (AST) profiles validated resistant pathogens, which were then proteomic profiled to investigate CS between colistin and meropenem at both phenotypic and proteome levels.

**Results:**

AST of developed panels revealed that colistin-resistant pathogens became sensitive to meropenem during adaptation to colistin, representing CS, which was validated by the emergence of colistin sensitivity when colistin-resistant *K. pneumoniae* adapted against meropenem. Proteomic analysis of the resistant panels identified a total of 3,126 proteins, including 23 and 5 differentially expressed proteins (DEPs) in colistin- and meropenem-resistant pathogens, respectively. Among these, 12 proteins (*phoP*, *arnB/arnD/arnT*, and *pmrD/pmrA*) (associated with two components: system regulator and antibiotic resistance); *degP*, *kdpB*, *maeA*, f*rmR*, *fhuC*, and *entF* (responsible for virulence and stress adaptation); and *fhuC*, *entF*, and *amn* (required for iron acquisition and persistence) showed altered pattern matching along with CS, that is, upregulated during emergence of colistin resistance along with meropenem sensitivity and conversely downregulated during emergence of colistin sensitivity along with meropenem-resistance in colistin-1x resistant *K. pneumoniae*.

**Conclusion:**

This study, for the first time, developed a unique panel of colistin- and meropenem-resistant *K. pneumoniae* cells representing the early, mid, and late stages of AMR emergence. The simultaneous emergence of CS for meropenem while developing resistance against colistin among the panels may pave the way to devise antibiotic cycling and/or co-dosing approaches, and modulation of the expression of the identified 12 potential proteomic biomarkers may be utilized to regulate the emergence of CS for meropenem in colistin-resistant pathogens for combating colistin-resistant infections.

## 1 Introduction

Antimicrobial resistance (AMR) has become one of the biggest global public health challenges, causing an estimated >10 million deaths per year by 2050 (Naghavi et al., 2024). It is among the top 10 global public health threats, attributed to the abuse, misuse, and overuse of antibiotics in clinical and agricultural settings (Elbehiry et al., 2025; WHO, 2026). The problem of AMR is quite serious in low- and middle-income countries (LMICs) in comparison to higher income countries (HICs) and is associated with poor healthcare infrastructure, inefficient diagnostic and surveillance networks, and limited treatment options (Pokharel et al., 2024; Davis et al., 2025).

Difficult-to-treat antibiotic-resistant *Enterococcus faecium, Staphylococcus aureus*, *K. pneumoniae*, *Acinetobacter baumannii*, *Pseudomonas aeruginosa*, and *Enterobacter* species (*ESKAPE* pathogens) are the major contributors of AMR-associated global deaths (Zeng et al., 2026). Of these, *K. pneumoniae* is among the top threats, causing pneumonia, bloodstream infections, urinary tract infections, and wound infections, particularly in hospitalized and immunocompromised patients (Kandemir et al., 2026). The ability of *K. pneumoniae* to rapidly ‘adapt’ and ‘escape’ the bactericidal activity of multiple classes of antibiotics, including last-resort (colistin) and carbapenems (meropenem/imipenem), has made its treatment difficult, resulting in prolonged hospital stays, increased healthcare costs, and high morbidity and mortality, and thus, immediately demands alternative and effective therapeutics and infection control strategies (Xie et al., 2026; Li et al., 2026; Ding et al., 2025).

Since *K. pneumoniae* is developing multidrug-resistance/extensively drug-resistance (MDR/XDR) faster than the antibiotic development pipeline, recovery of the huge investments made in the discovery and development of antimicrobials by the pharmaceutical industry (~1–1.7 billion USD) is becoming very difficult, and as a result, industries are losing their interest in the domain (Gargate et al., 2025; Skender, 2024). Thus, understanding the mechanisms of emergence of AMR has become critical for devising effective strategies to combat this global health threat. It is learned that resistance does not emerge randomly but follows defined evolutionary trajectories driven by genetic mutations, gene transfer, and adaptive regulatory molecular networks (MacLean et al., 2026; Nusrat et al., 2025). Alterations at the proteome level are among the major drivers and easy-to-detect indicators of such adaptive evolution. These adaptive molecular/proteomic changes enable bacteria to survive under the selective pressure of antimicrobials and may confer cross- or co-resistance to multiple drug classes (Darby et al., 2023; Pan et al., 2025; Duncan et al., 2023).

High-priority emerging domains for exploring alternative therapeutics primarily include collateral sensitivity (CS) (Hernando-Amado et al., 2023). CS is the mechanism by which pathogens become resistant to one (or one class of) antibiotic(s), but simultaneously become sensitive to other (or other classes of) antibiotic(s). Identifying antibiotics that show CS as pathogens become resistant to key antibiotics, such as colistin and meropenem, can save significant time and resources for the discovery and development of novel antimicrobials. The clinical implications of CS, that is, supplying prioritized rational therapies such as the sequential or concurrent deployment of reciprocal CS antibiotic pairs to combat AMR (Guo et al., 2024; Sanz-García et al., 2023; Maddamsetti et al., 2024), have been reported in bacterial pathogens. However, to date, only one study has reported the utility of the collateral effects between tetracycline and aminoglycoside resistance in carbapenem-resistant *K. pneumoniae* (Ma et al., 2023). More specifically, no study has explored the molecular mechanism mediating CS in carbapenem-resistant *K. pneumoniae* (for the last-resort “colistin” and the latest-generation “meropenem” antibiotics) to date. Therefore, this study extensively explored the molecular adaptation of *K. pneumoniae* to confer resistance to colistin and the emergence of CS to meropenem in laboratory-evolved colistin-resistant *K. pneumoniae* at the protein level, to identify protein biomarkers that contribute to the development of resistance and/or mediate CS and could be leveraged to combat AMR.

## 2 Materials and methods

### 2.1 Bacterial strains, media, and antibiotics

*K. pneumoniae* (ATCC 33495; ampicillin-resistant) and carbapenem-resistant bloodstream clinical isolates of *K. pneumoniae* were precultured aerobically in Mueller-Hinton Broth (MHB)/Mueller-Hinton Agar (MHA) (Difco™) overnight at 37 °C and 250 rpm, followed by fresh inoculation in MHB (1:500 v/v) until the mid-exponential phase of growth (~4 h post-inoculation) was achieved (Kumar et al., 2022). For the adaptive laboratory evolution of antibiotic resistance, freshly aliquoted cells were grown either with or without antibiotic pressure. Colistin (Sigma Catalog No. C1511-10MU) and meropenem (Sigma Catalog No. PHR1772-500MG) stock solutions were prepared in autoclaved Milli-Q water and dimethyl sulfoxide (DMSO), respectively, and stored at −20 °C for 3–5 days.

### 2.2 Minimum inhibitory concentration (MIC) evaluation via broth microdilution (BMD) and E-strips method

The 34th M100 Clinical and Laboratory Standards Institute (CLSI) 2024 recommendations for broth microdilution (BMD) were used to determine the minimum inhibitory concentrations (MICs) of colistin and meropenem against the bacterial strains (Alquait et al., 2026). In short, bacterial pathogens were cultured for 12–16 h at 37 °C and 250 rpm. To obtain the exponential-phase culture (OD, ~0.4–0.6), the overnight culture was diluted 1:1000 (v/v) in fresh 5 mL MHB and incubated at 37 °C and 250 rpm for 2–2.5 h. Following that, 96-well round-bottom plates (Coring® Catalog No. 3799) were seeded with antibiotics in accordance with CLSI guidelines at a cell density (OD-based measurement of cell number) of 10^6^ cells/mL. Additionally, a bacterial lawn was produced on MHA plates using a bacterial culture with an absorbance of around 0.4–0.6 with a sterile cotton swab (Hi-media) for E-strip-based MIC measurement (concentration gradient of colistin [Hi-media EM020-30ST] and meropenem [Hi-media EM080-30ST]) (Alquait et al., 2026). E-strip-exposed plates were incubated for 12–16 h at 37 °C. The MIC was determined by measuring the zone of inhibition (mm), and the ChemiDoc™ MP Imaging System (Bio-Rad) was used to record the images.

### 2.3 Development of antibiotic-resistant *Klebsiella pneumoniae* panel and its validation

The BMD was used to determine the MICs for meropenem and colistin. After being subjected to 0.25x MIC of antibiotics (sub-lethal dose), *K. pneumoniae* cells were sub-cultured until they adapted and attained O.D. ≥ 0.6 (in ≥3 consecutive biological replicates), comparable to the parent strain control/wild type (WT) over a 4 h incubation period (referred to as 0.25x-adapted cells). The adapted cells were stocked (regarded as 0.25xMIC adapted) and then transferred to the next level of increased dose (i.e., 0.5xMIC; regarded as 0.25xMIC adapted). The process was followed until cells adapted to 1xMIC (regarded as 0.25xMIC adapted). This procedure was carried out independently for both the antibiotics meropenem and colistin. Two panels of modified cells (0.25x, 0.5x, and 1xMIC resistant) were developed; the panel of developed resistant cells was MIC validated using absorbance and E-strip methods as described above (Section 2.2).

### 2.4 Pathogen identification and antimicrobial susceptibility test characterization

Matrix-Assisted Laser Desorption/Ionization Time-of-Flight (MALDI-TOF, bioMerieux serial number: 51224) was used to validate the identity of the adapted bacterial strains cultured on MHA. In accordance with European Committee on Antimicrobial Susceptibility Testing (EUCAST) recommendations, these samples were simultaneously tested for antibiotic susceptibility using the VITEK-2 compact system (bioMerieux serial number VK2C17824) utilizing AST Card No. 407. The AST data were obtained in document form, used for comparison analysis, and interpreted in accordance with EUCAST rules (Park et al., 2026).

### 2.5 Proteomics sample preparation and protein extraction

The panels of developed resistant *K. pneumoniae* cells representing the parent strain (0x [WT]), resistant at 0.25xMIC, 0.5xMIC, and 1xMIC (each for colistin and meropenem) were cultured with the respective antibiotic concentrations and harvested in the mid-exponential phase of culture (after 4 h of incubation). The cells were washed twice with cold phosphate-buffered saline (PBS), and the pellets were stored at −80 °C until and used. Notably, 50 μg of proteins were denatured with 8 M urea at a concentration of 1 mg/mL for *in situ* trypsin digestion. After reduction with 10 mM dithiothreitol (DTT) for up to 1 h at 37 °C, alkylation with 20 mM indole-3-acetic acid (IAA) was performed in the dark. The concentration was diluted to 2 M by adding 50 mM ammonium bicarbonate to the sample mixture. Trypsin was used to break down proteins at a 1:20 ratio overnight at 37 °C. After digestion, the peptide mixture was vacuum-concentrated, desalted, and acidified with formic acid.

### 2.6 Liquid chromatography–tandem mass spectrometry (LC–MS/MS) conditions

Trypsin-digested samples were desalted offline by a C18 column using the Orbitrap Exploris™ 480 Mass Spectrometer (Thermo Scientific, Bremen, Germany) before being loaded into a trap. The high-performance liquid chromatography (HPLC) mobile phases were acetonitrile/formic acid (80/0.1%) and water/formic acid (100/0.1%). A flow rate of 300 nL/min was used to inject 500 ng of sample into the column. The following gradient conditions were used to achieve analytical separation: 6% B for the first 2 min, then a linear gradient to 31% B for 58 min, and finally another linear gradient to 44% B for 30 min. The gradient was raised to 100% B for 1 minute and maintained for 9 min after the peptide elution window.

### 2.7 Database search for protein identification

Proteome Discoverer software with the CHIMERYS algorithm and INFERYS rescoring was used to identify proteins in the samples by searching the UniProt database for *K. pneumoniae* (taxon ID: 573). The entire proteomics dataset produced in this study, including raw LC–MS/MS files, protein identification results, and related experimental metadata, has been deposited in the Indian Biological Data Center (IBDC), under project ID: IPD5407, to promote transparency and reproducibility.

### 2.8 Statistical analyses

The identified proteins were analyzed using MetaboAnalyst 5.0 to determine *p*-values (≤0.05: significant) (used to evaluate the statistical significance of these changes across biological replicates) and the log2 fold change (Log2FC) (used to determine the magnitude and direction of changes in protein abundance) in comparison to the control (WT). To identify the important proteins in each scenario, all potential comparison groups (Supplementary Table S1) were established. Volcano and UpSet plots were used to statistically and functionally analyze the list of important proteins under various situations. Using GraphPad Prism version 10.1.0 (GraphPad Software, San Diego, CA, USA), two-way analysis of variance (ANOVA) *post hoc* multiple comparison tests (such as Sidak’s) were used to statistically compare all significant proteins based on those common to both ATCC and clinical strains.

### 2.9 Key differentially expressed proteins (DEPs) heatmap and protein–protein interaction (PPI)

GraphPad Prism version 10.1.0 (GraphPad Software, San Diego, CA, USA) was used to plot a heatmap based on the abundance of 12 key differentially expressed proteins (DEPs). The STRING database (Version: 12.0) was used to create protein–protein interaction (PPI) networks. Text mining, experiments, databases, co-expression, neighborhood, gene fusion, and co-occurrence were used to select interactions. Network Parameters used: Number of nodes: 12, Number of edges: 8, Average node degree: 1.33, Average local clustering coefficient: 0.375, PPI enrichment *p*-value: 3.26e-05, and medium confidence 0.400. For network visualization in Cytoscape version 3.10.2, proteins were clustered by function based on an extensive literature search (Figure 1).

**Figure 1:**
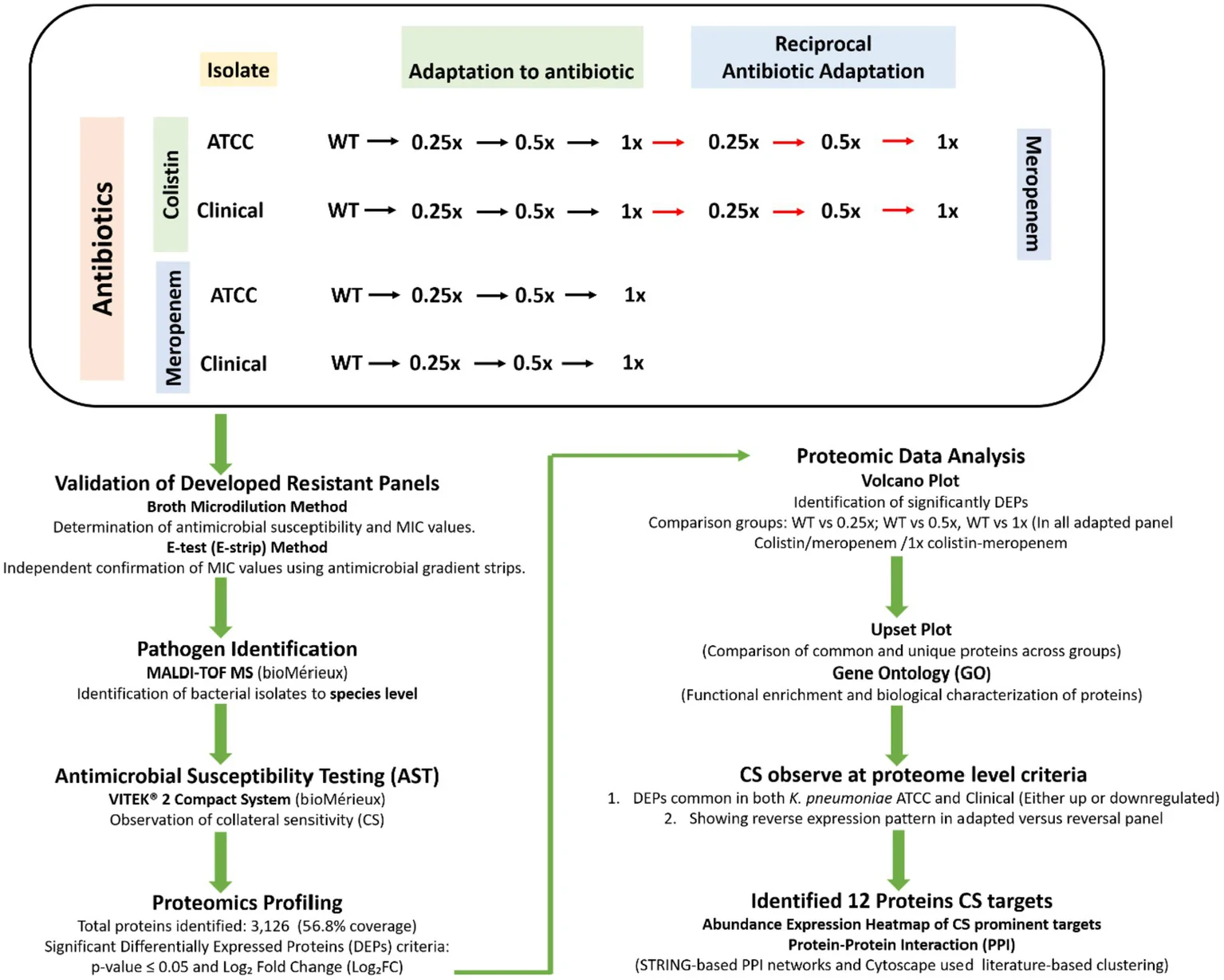
A schematic representation of the methodological framework and experimental workflow used in this study.

## 3 Results

### 3.1 Development of panels of colistin and meropenem resistance *Klebsiella pneumoniae*

Panels of gradually adapted *K. pneumoniae* (ATCC and clinical strains) were generated *in vitro* using gradually increasing sub-lethal doses (0.25x, 0.5x, and 1xMIC) of colistin and meropenem. Briefly, *K. pneumoniae* cells (10^6^ cells/mL) were repeatedly exposed to sub-lethal antibiotic concentrations (Figure 2) till they achieved cutoff growth (absorbance [OD, ~0.4–0.6]) after 4 h of culture, a comparable OD to that of the control/WT strain, over three consecutive cultures. Once the cells reached the cutoff OD, they were exposed to the next level of increased antibiotic concentration. Following this workflow, cells resistant to 0.25x, 0.5x, and 1xMIC were developed. Generating these panels took ~90 days for colistin, ~110 days for meropenem in *K. pneumoniae*, and ~60 days for meropenem in colistin-resistant *K. pneumoniae*, for a cumulative time of ~260 days, with the pathogens well segregated to avoid cross-contamination.

**Figure 2:**
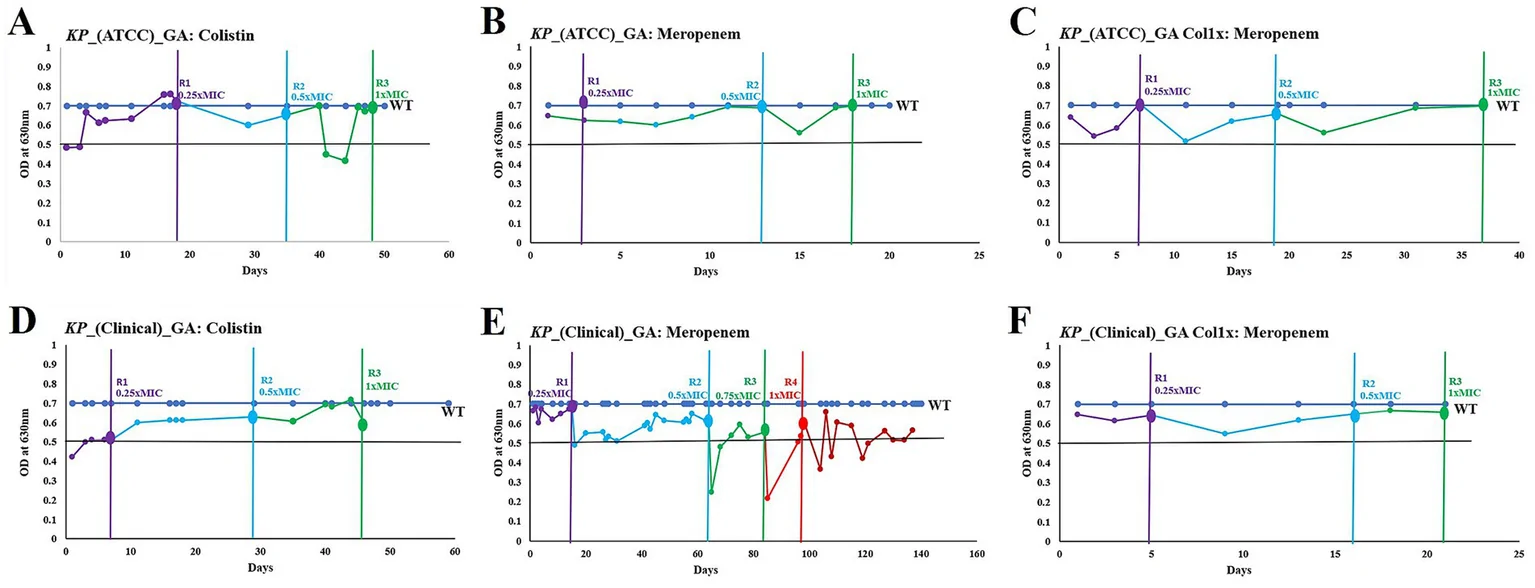
*In vitro* developed panels of colistin and meropenem resistance *Klebsiella pneumoniae*. **(A)** *K. pneumoniae* (ATCC) exposed to colistin; **(B)** *K. pneumoniae* (ATCC) exposed to meropenem; **(C)** 1x colistin-adapted *K. pneumoniae* ATCC exposed to meropenem; **(D)** *K. pneumoniae* (clinical) exposed to colistin; **(E)** *K. pneumoniae* (clinical) exposed to meropenem; **(F)** 1x colistin-adapted *K. pneumoniae* (clinical) exposed to meropenem.

More specifically, for *K. pneumoniae* (ATCC), adaptation to colistin took almost 18 days to reach 0.25xMIC, 22 days to reach 0.5x from 0.25xMIC-adapted cells, and then 8 days to reach 1x from 0.5xMIC-adapted cells (Figure 2A). For *K. pneumoniae* (clinical), it took 8 days to adapt to 0.25xMIC, 21 days to adapt to 0.5xMIC from 0.25xMIC-adapted cells, and additional 21 days to adapt to 1xMIC from 0.5xMIC-adapted cells (Figure 2D).

In the case of Meropenem, *K. pneumoniae* (ATCC) adaptation took 7 days to reach 0.25xMIC, 5 days to reach 0.5xMIC from 0.25xMIC-adapted cells, and 5 days to reach 1xMIC from 0.5xMIC-adapted cells (Figure 2B). For *K. pneumoniae* (clinical), it took 15 days to adapt to 0.25xMIC, 49 days to adapt to 0.5xMIC from 0.25xMIC-adapted cells, 20 days to adapt to 0.75xMIC from 0.5xMIC-adapted cells, and 14 days to adapt to 1xMIC from 0.75xMIC-adapted cells (Figure 2E).

To investigate the effect of CS, 1xMIC colistin-adapted *K. pneumoniae* (ATCC and clinical) cells were gradually adapted to meropenem (Figures 2C,F). In the case of 1xMIC colistin-adapted *K. pneumoniae* (ATCC), it took 7 days to adapt to 0.25xMIC, 12 days to adapt to 0.5x from 0.25xMIC-adapted cells, and 19 days to adapt to 1xMIC from 0.5xMIC-adapted cells (Figure 2C). For 1xMIC colistin-adapted *K. pneumoniae* (clinical), it took 5 days to adapt to 0.25xMIC, 11 days to adapt to 0.5xMIC from 0.25xMIC-adapted cells, and 5 days to adapt to 1xMIC meropenem from 0.5xMIC-adapted cells (Figure 2F). Overall, adaptation to different antibiotic concentrations showed that adaptation to 0.5xMIC took longer than to other concentrations (0.25x and 1xMIC, etc.).

### 3.2 Validation of developed resistance *Klebsiella pneumoniae* panels: cross-contamination and pathogen identity

Colistin- and meropenem-resistant *K. pneumoniae* (ATCC and clinical strains), along with 1xMIC colistin-resistant *K. pneumoniae* adapted to meropenem (ATCC and clinical strains), were subjected to pathogen identification using MALDI-TOF MS to rule out cross-contamination in the developed resistant panels. All samples were identified as *K. pneumoniae* (confidence value >99.9%) (Supplementary Table S2).

### 3.3 Validation of colistin, meropenem, and 1xMIC colistin-resistant meropenem-adapted panel of *Klebsiella pneumoniae* and observing collateral sensitivity

The developed resistant *K. pneumoniae* cells were validated by culturing them in the presence and absence of antibiotics. For absorbance-based validation, for example, the 0.25xMIC colistin-adapted cells were grown in MHB at a colistin concentration of 0.25xMIC, and control/WT cells were grown in MHB at a similar 0.25x colistin concentration. A similar method was followed for the other adapted cells in the developed resistant panel. The WT strain showed no growth (Supplementary Figures S2A–F) when exposed to sub-lethal MIC (0.25x, 0.5x, and 1x) doses, except in the case of meropenem, where WT was only sensitive to the 1xMIC (Figures 3A–E). However, the adapted cells (0.25x, 0.5x, and 1x adapted) were able to grow when supplemented with antibiotic concentrations similar to those with which they were adapted (Figures 3A–E). Similar results were observed in the E-strips assay, where WT showed no growth, in contrast to the adapted cells.

**Figure 3:**
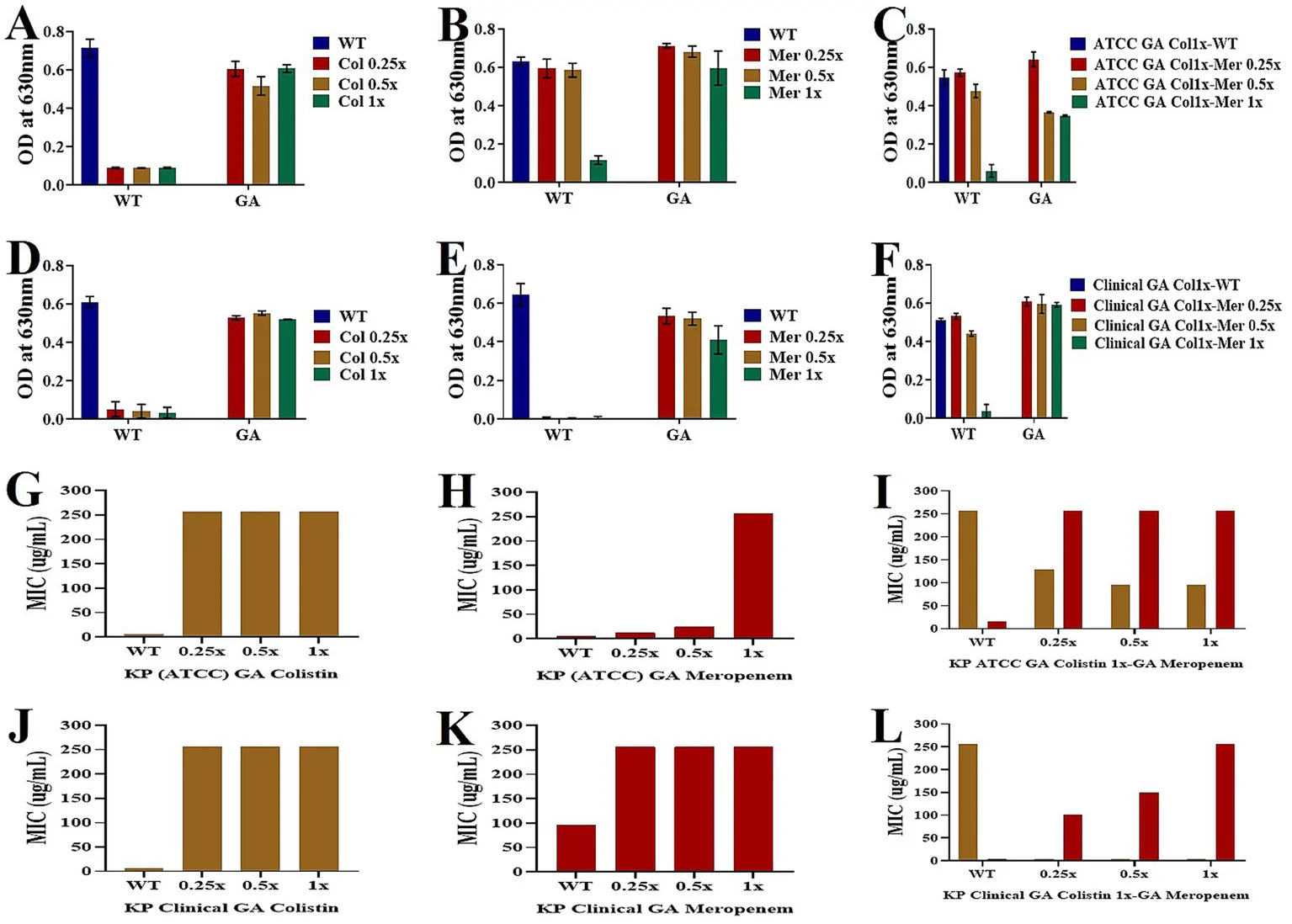
Validation of developed resistant panels. Absorbance-based validation: 0.25x, 0.5x, and 1x-adapted *Klebsiella pneumoniae* **(A)** colistin-adapted *K. pneumoniae* (ATCC); **(B)** meropenem-adapted *K. pneumoniae* (ATCC); **(C)** meropenem-adapted 1x colistin-resistant *K. pneumoniae* (ATCC); **(D)** colistin-adapted *K. pneumoniae* (Clinical); **(E)** meropenem-adapted *K. pneumoniae* (Clinical); **(F)** meropenem-adapted 1x colistin-resistant *K. pneumoniae* (Clinical). E-strips assay-based validation 0.25x, 0.5x, and 1x-adapted *K. pneumoniae* **(G)** colistin-adapted *K. pneumoniae* (ATCC); **(H)** meropenem-adapted *K. pneumoniae* (ATCC); **(I)** meropenem-adapted 1x colistin-resistant *K. pneumoniae* (ATCC); **(J)** colistin-adapted *K. pneumoniae* (Clinical); **(K)** meropenem-adapted *K. pneumoniae* (Clinical); **(L)** meropenem-adapted 1x colistin-resistant *K. pneumoniae* (Clinical).

Furthermore, in the case of panels developed to study the effect of CS, that is, 1xMIC colistin-adapted *K. pneumoniae* (ATCC and clinical strains) adapted to meropenem, a slightly different and interesting pattern was observed. When 1xMIC colistin-adapted *K. pneumoniae* (ATCC) was exposed to meropenem, the cells were sensitive to meropenem (Figure 3C). However, when the 1xMIC colistin-adapted *K. pneumoniae* (clinical) cells were adapted to meropenem (Figure 3F), they became sensitive to colistin, confirming CS.

These observations were also confirmed by E-strip based assays (Figure 3; Supplementary Figure S2). *K. pneumoniae* (ATCC and clinical strains) WT strains showed sensitivity to colistin, and a higher zone of inhibition (<MIC) was observed. However, upon gradual adaptation, they developed resistance (0.25x, 0.5x, and 1xMIC) toward colistin; thus, the zone of inhibition decreased, and MIC increased (Figures 3G,J). A similar trend was observed when *K. pneumoniae* (ATCC and clinical strains) WT strains were adapted to meropenem (Figures 3H,K). However, in the case of *K. pneumoniae* (ATCC) adaptation toward meropenem, a gradual decrease in the zone of inhibition (gradual increase in MIC) was observed (Figure 3H), which was not exactly similar to other adapted cells, as they became saturated upon adaptation to a 0.25xMIC concentration. No gradual effect was observed (Figures 3G,J,K). The E-strip assay of 1xMIC colistin-adapted *K. pneumoniae* (ATCC) adaptation toward meropenem showed a gradual increase in sensitivity toward colistin and resistance to meropenem (Figure 3I). However, in the case of *K. pneumoniae* (clinical), 100% sensitivity to colistin was achieved early with 0.25x meropenem-adapted cells, and a gradual pattern of resistance development to meropenem was observed (Figure 3L).

### 3.4 Validation of observed collateral sensitivity using automated antibiotic susceptibility testing (AST)

Panels were developed and used to validate AST profiles on the VITEK-2 compact system with an AST card (N-407), in which MICs were calculated automatically and interpreted according to EUCAST guidelines (Supplementary Table S3). Analysis of the AST profiles of *K. pneumoniae* (ATCC and clinical) panels revealed that all cells were resistant to cefepime (cell wall), ceftizoxime (cell wall), ampicillin/sulbactam (cell wall), chloramphenicol (protein synthesis), levofloxacin (nucleic acid), tetracycline (protein synthesis), and doxycycline (protein synthesis). Interestingly, a collateral dynamic effect was also observed in the AST profile of colistin/polymyxin B (cell membrane) adapted and 1xMIC colistin-resistant *K. pneumoniae* adapted to meropenem (cell wall), supporting the E-strip assay data (Figure 4; Supplementary Figures S3A–F). The control/WT cells, which were sensitive to colistin, gradually became resistant to colistin. When reversal adaptation was done, that is, adaptation of 1xMIC colistin-resistant cells to meropenem, the cells lost resistance to colistin and became sensitive, thus showing CS.

**Figure 4:**
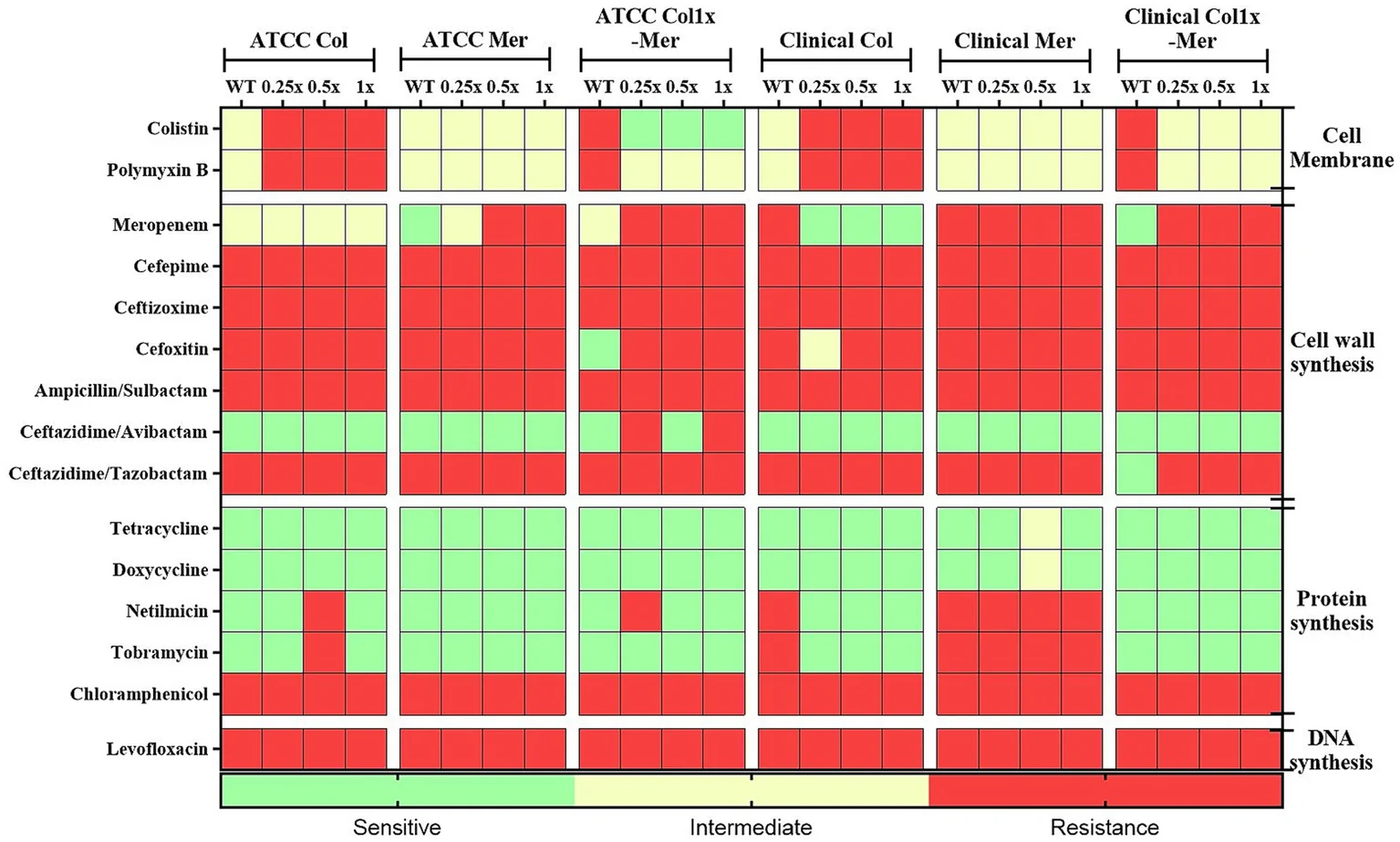
Antimicrobial susceptibility (AST) profile of resistant *Klebsiella pneumoniae* cells generated using an automated VITEK-2 system. Horizontal gaps among rows denote antibiotic categories based on modes of action. Vertical gaps in the columns denote different resistant panels such as ATCC/clinical colistin, meropenem, and 1xMIC colistin-resistant adapted to meropenem.

### 3.5 Comparative proteomic profiling of colistin, meropenem, and 1xMIC colistin-resistant adapted to meropenem *Klebsiella pneumoniae*

Label-free quantitative proteomic profiles were established to investigate proteomic alterations during the evolution of antibiotic resistance to colistin and meropenem in 1xMIC colistin-resistant meropenem-adapted *K. pneumoniae* cells. A total of 3,126 proteins (representing ~56.8% of the predicted *K. pneumoniae* “taxon ID 573” proteome-UniProt reference proteome; ~5,500 predicted proteins) were identified under all conditions. The list was used to identify differentially expressed proteins (DEPs) across comparison groups (Supplementary Table S1) using a cutoff of log2 fold change (Log2FC) and *p*-value ≤ 0.05.

Colistin-adapted *K. pneumoniae* (ATCC): In 0.25xMIC-adapted cells, 36 DEPs (23 upregulated and 13 downregulated) were identified; this increased to 258 DEPs (194 upregulated and 64 downregulated) in 0.5xMIC-adapted cells and to 247 DEPs (147 upregulated and 100 downregulated) in 1xMIC-adapted cells. 9 proteins were found to be common under all conditions (Figure 5; Table 1 (panel A); Supplementary Figure S4A–C; Supplementary Excel S1).

**Figure 5:**
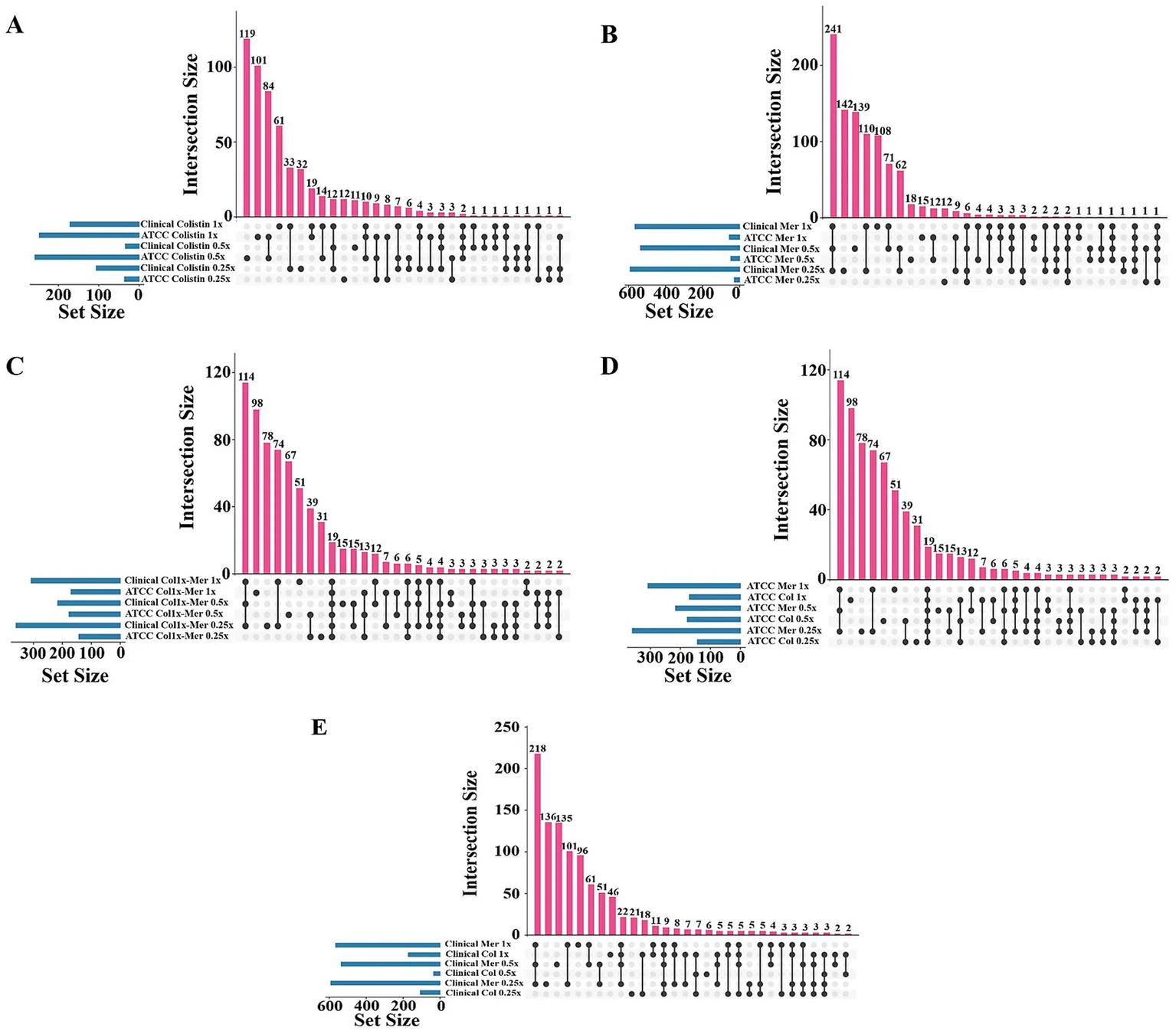
UpSet plots of DEPs identified in **(A)** Colistin adaptation; **(B)** Meropenem adaptation; **(C)** 1x colistin-resistant meropenem adaptation; **(D)** ATCC strain adaptation to either colistin or meropenem; **(E)** Clinical strain adaptation to either colistin or meropenem. Set size denotes the number of DEPs in each condition.

**Table 1:** DEPs identified in gradually adapted panels of *K. pneumoniae*.

Antibiotics	0.25xMIC adapted	0.5xMIC adapted	1xMIC adapted
Panel A: *K. pneumoniae* (ATCC) DEPs			
Colistin	36 (23 upregulated and 13 downregulated)	258 (194 upregulated and 64 downregulated)	247 (147 upregulated and 100 downregulated)
	9 common (0 upregulated, 4 downregulated, and 5 non-significant)		
Meropenem	28 (12 upregulated and 16 downregulated)	47 (33 upregulated and 14 downregulated)	55 (43 upregulated and 12 downregulated)
	1 common (0 upregulated, 0 downregulated, and 1 non-significant)		
1xMIC colistin-resistant adapted to meropenem	143 (114 upregulated and 29 downregulated)	177 (103 upregulated and 74 downregulated)	170 (147 upregulated and 23 downregulated)
	38 common (2 upregulated, 22 downregulated, and 14 non-significant)		
Panel B: *K. pneumoniae* (Clinical) DEPs			
Colistin	106 (19 upregulated and 87 downregulated)	34 (7 upregulated and 27 downregulated)	171 (66 upregulated and 105 downregulated)
	17 common (1 upregulated, 9 downregulated, and 7 non-significant)		
Meropenem	591 (338 upregulated and 253 downregulated)	535 (296 upregulated and 239 downregulated)	565 (328 upregulated and 237 downregulated)
	255 common (50 upregulated, 56 downregulated, and 149 non-significant)		
1xMIC colistin-resistant adapted to meropenem	360 (291 upregulated and 69 downregulated)	216 (151 upregulated and 65 downregulated)	308 (221 upregulated and 87 downregulated)
	154 common (22 upregulated, 57 downregulated, and 75 non-significant)		

DEPs colistin	DEPs meropenem	1xMIC colistin-resistant adapted to meropenem
Panel C: Common in ATCC and clinical strains		
23 (15 upregulated and 8 downregulated)	5 (4 upregulated and 1 downregulated)	54 (6 upregulated and 48 downregulated)

DEPs in ATCC	DEPs in Clinical
Panel D: Common in colistin and meropenem	
20 (17 upregulated and 3 downregulated)	23 (6 upregulated and 17 downregulated)

Differentially expressed proteins (DEPs) identified in *Klebsiella pneumoniae* for colistin (A), meropenem (B), common DEPs independent of strains (C), and common DEPs independent of antibiotics (D).

Meropenem-adapted *K. pneumoniae* (ATCC): In 0.25xMIC-adapted cells, a total of 28 DEPs (12 upregulated and 16 downregulated) were observed, increasing to 47 DEPs (33 upregulated and 14 downregulated) in 0.5xMIC-adapted cells and to 55 DEPs (43 upregulated and 12 downregulated) in 1xMIC-adapted cells. Only 1 protein was found to be common under all conditions (Figure 5; Table 1 (panel A); Supplementary Figure S4D–F; Supplementary Excel S1).

1xMIC colistin-resistant adapted to meropenem *K. pneumoniae* (ATCC): In 0.25xMIC-adapted cells, a total of 143 DEPs (114 upregulated and 29 downregulated) were observed, increasing to 177 DEPs (103 upregulated and 74 downregulated) in 0.5xMIC-adapted cells and 170 DEPs (147 upregulated and 23 downregulated) in 1xMIC-adapted cells. A total of 38 proteins were found to be common under all conditions (Figure 5; Table 1 (panel A); Supplementary Figures 4G–I; Supplementary Excel S1).

Colistin-adapted *K. pneumoniae* (clinical): In 0.25x-adapted cells, a total of 106 DEPs (19 upregulated and 87 downregulated) were found, whereas 34 DEPs (7 upregulated and 27 downregulated) were found in 0.5xMIC-adapted cells and 171 DEPs (66 upregulated and 105 downregulated) were found in 1xMIC-adapted cells. A total of 17 proteins were found to be common under all conditions (Figure 5; Table 1 (panel B); Supplementary Figure S5A–C; Supplementary Excel S2).

Meropenem-adapted *K. pneumoniae* (clinical): In 0.25xMIC-adapted cells, a total of 591 DEPs (338 upregulated and 253 downregulated), 535 DEPs (296 upregulated and 239 downregulated) were found in 0.5xMIC-adapted cells, and 565 DEPs (328 upregulated and 237 downregulated) were found in 1xMIC-adapted cells. A total of 255 proteins were found to be common under all conditions (Figure 5; Table 1 (panel B); Supplementary Figures S5D–F; Supplementary Excel 2).

1xMIC colistin-resistant adapted to meropenem *K. pneumoniae* (clinical): In 0.25xMIC-adapted cells, 360 DEPs (291 upregulated and 69 downregulated) were observed, whereas 216 DEPs (151 upregulated and 65 downregulated) were observed in 0.5xMIC-adapted cells and 308 DEPs (221 upregulated and 87 downregulated) in 1xMIC-adapted cells. A total of 154 proteins were found to be common under all conditions (Figure 5; Table 1 (panel B); Supplementary Figures S5G–I; Supplementary Excel S2).

Furthermore, the Protein Analysis Through Evolutionary Relationships (PANTHER) enrichment analysis tool was used to determine the molecular function trajectory of DEPs using Gene Ontology (GO) terms, and high catalytic activity (GO:0003824) was enriched in both ATCC and clinical strains. This suggests that biological systems are in a metabolically active state and undergo adaptive cellular physiological changes, which are often reflected by processes such as altered energy metabolism, stress response, and antibiotic resistance mechanisms [Supplementary Figures S6.1A–I for ATCC adapted and Supplementary Figures S6.2A–I for Clinical adapted].

High-priority DEPs involved in the development of resistance to colistin and meropenem, and 1xMIC colistin-resistant adapted to meropenem *K. pneumoniae*, were identified by applying the following criteria: (1) it should be differentially expressed in both *K. pneumoniae* (ATCC and clinical strains); (2) the expression pattern should either show a gradual upward or downward trend. Following this, a total of 23 DEPs potentially responsible for colistin resistance, 5 DEPs potentially responsible for meropenem resistance, and 54 DEPs potentially responsible for 1xMIC colistin-resistant adapted to meropenem *K. pneumoniae*, independent of the strains (ATCC or Clinical), were identified and included in further analysis (Table 1 (panels C, D); Supplementary Table S1; Supplementary Figures S7A–C). Furthermore, the DEPs potentially responsible for colistin resistance (at 0.25x, 0.5x, and 1x) and meropenem resistance (at 0.25xMIC, 0.5xMIC, and 1xMIC) were compared among strains, and a total of 20 DEPs in ATCC and 23 in clinical strains were identified as strain-specific DEPs potentially associated with the evolution of resistance among them (Supplementary Tables S7A–E; Supplementary Figures S7D,E). Interestingly, a single protein (SH3b domain-containing protein) was identified as differentially expressed (at 0.25x, 0.5x, and 1x) in both *K. pneumoniae* (ATCC) and *K. pneumoniae* (clinical), and independent of the antibiotics used to develop the resistance panels. The DEPs were studied in the published literature for their significance, cellular localization, and biological functions in the emergence of AMR (Supplementary Tables S7A–E; Supplementary Figure S8).

Furthermore, DEPs involved in collateral dynamics were identified (DEPs showing reverse expression between colistin-resistant cells and 1xMIC colistin-resistant cells adapted to meropenem). Altogether, 12 DEPs (*ymoA*, *kdpB*, *fhuC*, *phoP*, *arnB*, *pmrD/pmrA*, *arnD/arnT*, *entF*, *frmR*, *maeA*, *degP/degQ*, and *amn/*AMP nucleosidase) potentially associated with CS were identified (Figures 6, 7).

**Figure 6:**
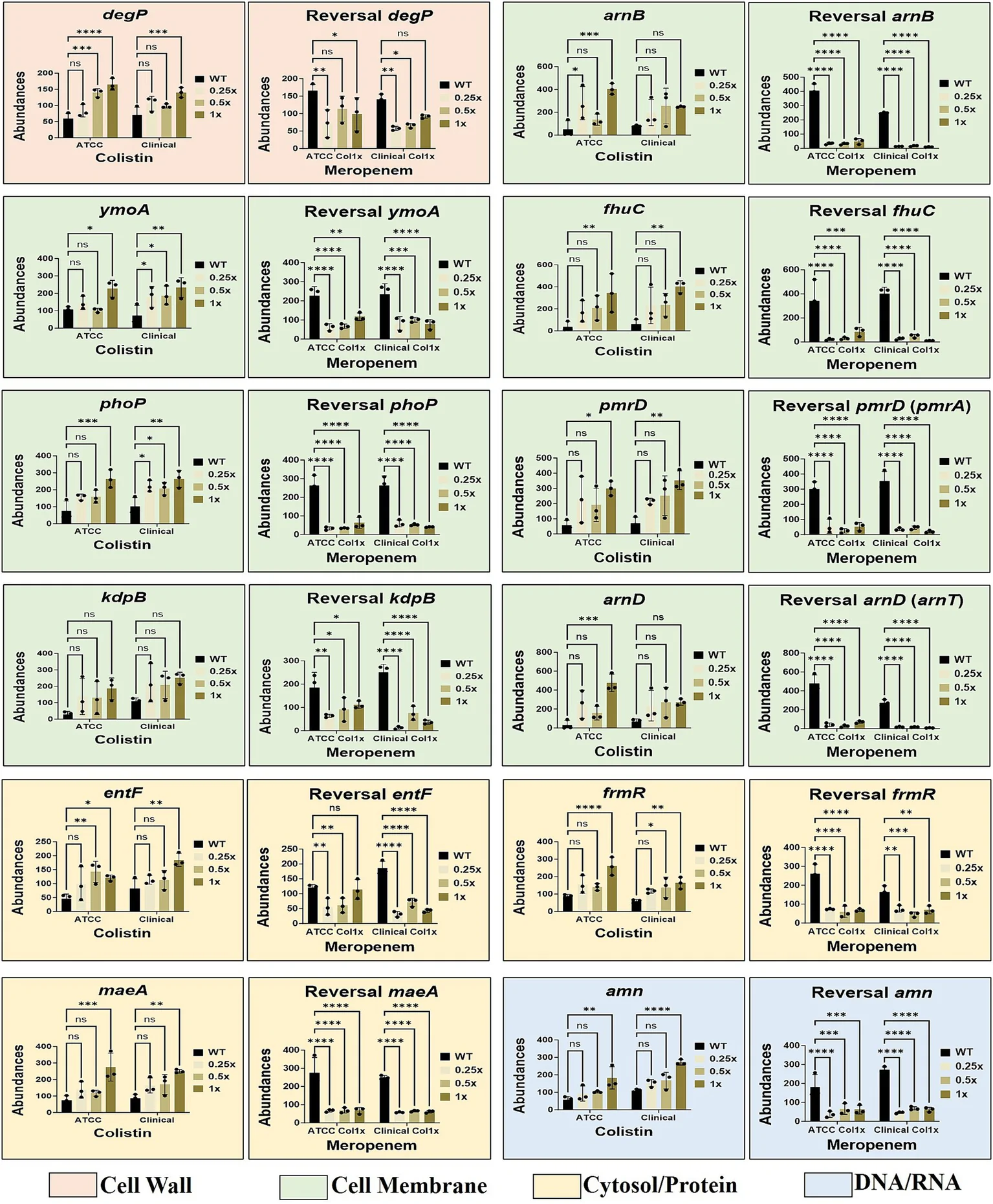
Proteomic abundance of 12 Key DEPs associated with collateral sensitivity (CS). DEPs were categorized and shown as per their localization within the *Klebsiella pneumoniae* cell.

**Figure 7:**
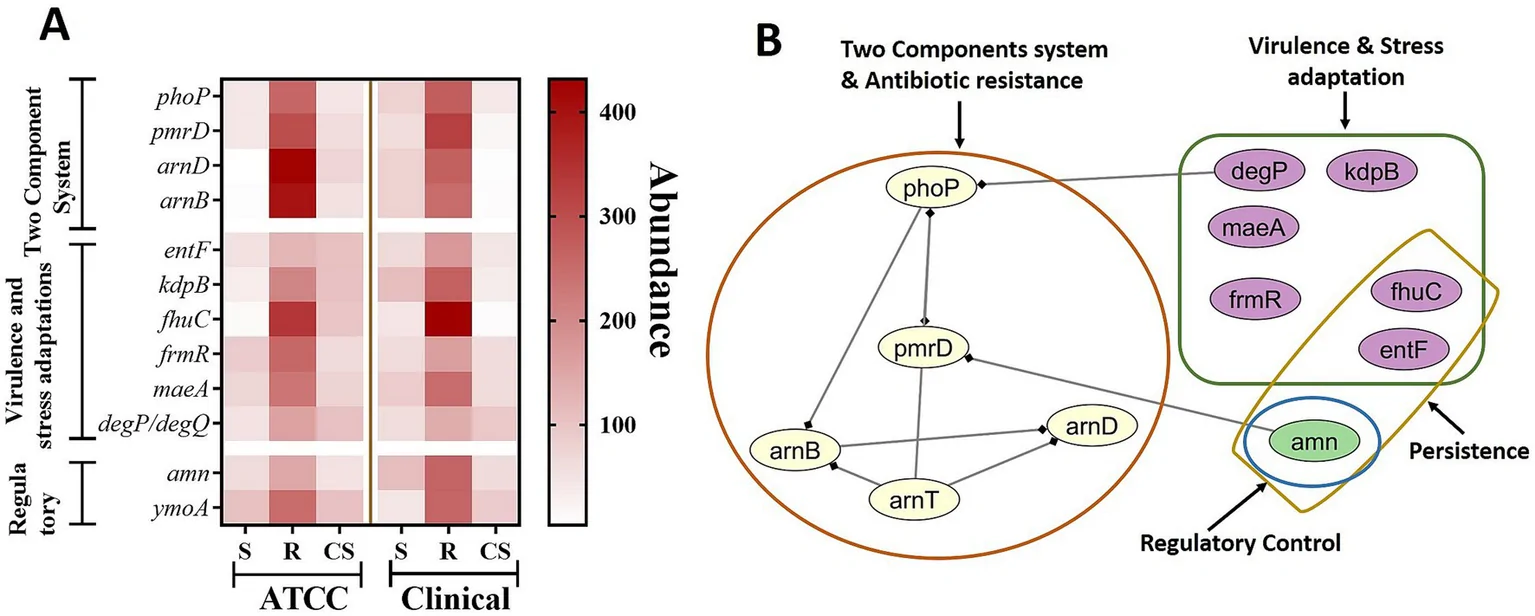
**(A)** Proteomic abundance of 12 key DEPs associated with collateral sensitivity was categorized into Two components systems, Virulence and stress adaptations, and regulatory controls in ATCC as well as Clinical (S-sensitivity and R-resistance, CS- collateral sensitivity). **(B)** Protein–protein interactions of DEPs clustered into biological functions in Cytoscape software version 3.10.2. Network Parameters: Number of nodes:12, Number of edges:8, Average node degree:1.33, Average local clustering coefficient:0.375, PPI enrichment *p*-value: 3.26e-05 and medium confidence 0.400. Each cluster represents a tightly interconnected group, such as two-component systems/antibiotics, virulence and stress adaptation, regulatory control, and persistence within the *K. pneumoniae* cell.

## 4 Discussion

Antimicrobial resistance (AMR) is one of the world’s greatest problems and is rapidly becoming a grave issue. It is the primary cause of mortality worldwide, mainly caused by *ESKAPE* pathogens. Among *ESKAPE* pathogens, *K. pneumoniae* is an egregious pathogen causing pneumonia, urinary tract infections, and bloodstream infections in hospitalized and immunocompromised patients, and it rapidly develops antibiotic resistance to survive. However, in the last few decades, the pace of new antibiotic discovery has declined. Taken together, this limits the availability of effective treatments for antibiotic-resistant pathogens. Hence, immediate attention is required to develop novel, effective, and/or alternative therapies. Potential options for alternative therapeutic discovery include bacteriophage therapy, antimicrobial peptides, probiotics, Clustered Regularly Interspaced Short Palindromic Repeats (CRISPR)-based antimicrobials, host-directed therapies, and collateral sensitivity (CS). However, since the need of the hour is a rapid and cost-effective solution, the present study focused on identifying CS antibiotic(s) for colistin-resistant *K. pneumoniae*.

In this study, as a proof of concept, *K. pneumoniae* (ATCC and clinical strains) was induced to develop resistance to colistin (a last-resort antibiotic) and meropenem (a latest-generation antibiotic). Briefly, *K. pneumoniae* was gradually adapted to 0.25x, 0.5x, and 1xMIC of antibiotics, cross-contamination and identity were verified using MALDI-TOF, and resistance was validated using absorbance, E-strips, and Vitek 2-based assays. The adaptation of bacteria to 0.5xMIC was more time-consuming compared to other concentrations (0.25x and 1xMIC). 1xMIC population was selected as the endpoint of the adaptive laboratory evolution panel. Higher antibiotic concentrations (2x and 4xMIC) were not performed or incorporated in the present study since exposure above the MIC is expected to slow bacterial adaptation and emergence of AMR significantly.

Interestingly, compared with prevoius studies, adaptation to colistin and meropenem in our laboratory took longer and using this method. For example, adaptation of *Escherichia coli* to 21 different antibiotics by the agar plate method was reported to take almost 10 days (Imamovic and Sommer, 2013). Another study reported *in vitro* experimental adaptation of three different *K. pneumoniae* colistin-sensitive strains to colistin in 48 h for each strain (Aulin et al., 2021). Similarly, a 30-day adaptation protocol was reported for 6 *Listeria monocytogenes* and 2 *Listeria innocua* strains against sub-lethal benzalkonium chloride (fluoroquinolones and co-trimoxazole) (Macleod et al., 2025). However, a potential reason for the disparity in the time required for pathogens to adapt to different antibiotics might be the differential need among bacterial pathogens/isolates for new pathways to take over (facilitated by genome alteration) and for their stabilization to compensate for the disturbance of intracellular homeostasis.

The developed panel of cells was then AST profiled, and CS to meropenem was observed in colistin-resistant *K. pneumoniae*. Briefly, 1xMIC colistin-resistant *K. pneumoniae* ATCC/clinical strains were sensitive to meropenem, whereas 1xMIC meropenem-resistant *K. pneumoniae* ATCC/clinical strains were resistant to colistin. Exposure of 1xMIC colistin-resistant *K. pneumoniae* ATCC/clinical strains to meropenem reversed the phenotype; that is, they became sensitive to colistin. Several studies have investigated CS in various pathogens using different antibiotic combinations. In one study, increased sensitivity of aminoglycoside-resistant *K. pneumoniae* strains to tetracyclines was associated with alterations in bacterial membrane potential. In contrast, the increased sensitivity to aminoglycosides in tetracycline-resistant strains was associated with an unbalanced oxidation–reduction process (Ma et al., 2023). In high-throughput collateral sensitivity screening, 29 antibiotic-resistant *E. coli* were screened against 80 commercial antimicrobials, revealing multiple CS interactions. Based on the knowledge generated, co-dosing of ceftazidime with borrelidin A has been reported to slow the emergence of broader cephalosporin resistance (Liu et al., 2023). Accumulation of mutations, potentially over-producing the efflux pump MexCD-OprJ in ciprofloxacin-resistant *P. aeruginosa*, was shown to develop sensitivity against tobramycin (Hernando-Amado et al., 2023); vancomycin-resistant *E. faecium* was shown to develop sensitivity for pleuromutilin, potentially due to epistasis between the *van* gene cluster and *msrC*, encoding an ABC-F protein that protects bacterial ribosomes from antibiotic targeting (Li et al., 2022). Collateral effects in *E. coli* have also been shown previously using surveillance data from the Pathosystems Resource Integration Center (PATRIC) database (Zwep et al., 2021). Altogether, this establishes that experimental evolution of AMR induces significant accumulation of mutations in the genome, which can change the resistance and sensitivity patterns to antimicrobials (Lázár et al., 2014). However, studies exploring the CS pair(s) for colistin in *K. pneumoniae* remain unexplored.

Further proteomic profiling of the developed resistance panel facilitated sequential decoding and understanding of the molecular mechanisms underlying antibiotic resistance. Enrichment of catalytic activity in molecular function analysis suggests that the biological system is metabolically active or undergoes adaptive physiological changes often linked to energy metabolism, stress responses, or antibiotic resistance. Differentially expressed proteins (DEPs) associated with colistin (23 DEPs) and meropenem resistance (5 DEPs) were identified in both the ATCC and clinical strains. Of the 23 colistin-resistant proteins identified, 16 were known to be associated with antibiotic resistance (Llobet et al., 2011; Holmes et al., 2023; Benn et al., 2024; Xie et al., 2025). Similarly, two proteins (ompK26 and SH3b) out of five proteins from meropenem-resistant proteins were known to be associated with antibiotic resistance (Zhao et al., 2025). Furthermore, a single DEP-SH3b domain-containing protein was identified as responsible for the development of resistance, irrespective of the strain or antibiotic. SH3b maintains cell wall homeostasis and can indirectly impact antibiotic resistance by improving cell wall flexibility or by promoting adaptation under antibiotic stress (Xu et al., 2015; Mitkowski et al., 2019). Although several studies have examined SH3b in different pathogens (*S. aureus* and *Clostridium perfringens*), no one has reported it in *K. pneumoniae* so far. However, compared with the ATCC strain, clinical isolates of *K. pneumoniae* exhibited substantially more DEPs. This may be due to a preexisting MDR phenotype, likely influenced by prior antibiotic exposure and host-associated stressors, resulting in more extensive physiological and regulatory remodeling. However, further investigation and experimental validation are still required.

Notably, analysis of 1x MIC colistin-resistant meropenem-adapted panels identified 12 key DEPs showing CS. Among these, 7 DEPs (*ymoA*, *kdpB*, *fhuC*, *phoP*, *arnB, pmrD/pmrA*, and *arnD/arnT*) were localized at the cell membrane (associated with colistin resistance) involved with lipopolysaccharide modification; 3 DEPs (*entF*, *frmR*, and *maeA*) were localized in the cytosol and were involved in protein synthesis, amino acid metabolism, and iron acquisition; 1 DEP (*degP/degQ*) was associated with the cell wall along and 1 DEP (*amn/*AMP nucleosidase) with nucleoid. Functionally, of the 12, 4 DEPs (*phoP*, *pmrD*, *arnD*, and *arnB*) were associated with two-component regulatory systems and may play important roles in antibiotic resistance. In comparison, 6 DEPs (*entF*, *kdpB*, *fhuC*, *frmR*, *maeA*, and *degP/degQ*) were involved in virulence and stress adaptation. The remaining two proteins (*amn* and *ymoA*) were regulatory. The *amn*, *fhuC*, and *entF* have also been reported to be involved in persistence.

It is crucial to understand that *K. pneumoniae* acquires resistance through a variety of chromosomal and plasmid-modification pathways, altering the expression of multiple targets. Modifications in the *phoP/phoQ* and *pmrA/pmrB* two-component regulatory systems (*mgrB* inactivation, *crrAB* activation, *pmrD* regulation, and acquisition of plasmid-mediated mcr genes) have all been shown to increase lipid A modification and decrease colistin binding, enabling colistin resistance. Our proteomic analysis revealed a core group of 12 DEPs linked to CS that are consistent with this genetic architecture. These DEPs include *phoP*, *pmrD*, *arnD*, and *arnB*, which are well-known elements of the lipid A modification pathway (Sahayarayan et al., 2024; Liu et al., 2021; Wright et al., 2015). The DEPs were directly correlated with the emergence of resistance, upregulated the development of resistance to colistin, and were downregulated when meropenem was supplemented to reverse adaptation in colistin-resistant *K. pneumoniae*. The altered expression of identified key biomarkers in our study may also be due to the accumulation of mutations, which may help the pathogen attain CS between colistin and meropenem (Zwep et al., 2021; Lázár et al., 2014). Since clinical isolates of colistin-resistant *K. pneumoniae* are infrequent and are currently unavailable in clinical practice due to restricted and limited use of colistin, the identified DEPs will be worth exploring as targets for combating AMR over time. However, the limited availability of colistin-resistant *K. pneumoniae* also highlights the timely to increase efforts to identify its CS pair and devise an antibiotic-cycling/co-dosing approach to preserve colistin as a valuable resource for long-term and the future use. Altogether, our study is the first to investigate the mechanistic basis of the CS between colistin resistance and meropenem susceptibility in *K. pneumoniae*.

### 4.1 Limitations

The study was performed, implying an adaptive laboratory evolution model for *K. pneumoniae* only; other Gram-negative pathogens were not included. Further, the study focused solely on profiling of proteome alterations during the emergence of colistin- and meropenem-resistant strains, proposing validation of the observations using other clinical strains as well as other tools over time. Furthermore, the molecular processes identified do not establish direct functional causation. After all, they were largely based on proteome profiling and specific gene expression analyses.

## 5 Future scope

The study opens the following new study areas for investigation in the future:

(1) Only one pathogen (*K. pneumoniae*; ATCC and clinical strains) and two antibiotics, that is, colistin and meropenem, were included in this study. More pathogens (Gram-negative and Gram-positive) and clinical isolates can be included, along with multiple antibiotics.
(2) For a more comprehensive understanding of the emergence of AMR, other big data approaches like transcriptomics and metabolomics would be required to complement the proteomics data.
(3) The role of 12 DEPs can be validated by performing knockout/overexpression studies.
(4) At present, there are no FDA-approved inhibitors available for DEPs identified in this study and can be screened/ repurposed/designed in the future.

## 6 Conclusion

Our study demonstrates (1) the development of a unique panel of colistin- and meropenem-resistant *K. pneumoniae* cells representing early, mid and late stages of emergence of AMR, (2) observed CS for the meropenem in colistin-resistant *K. pneumoniae,* (3) established the proteome profile of colistin and meropenem-resistant *K. pneumoniae* cells that can help to understand the molecular mechanism of emergence of AMR and identify diagnostic biomarker and drug targets and most importantly (4) identified 12 proteins (*phoP*, *arnB/arnD/arnT*, *pmrD/pmrA*, *degP*, *kdpB*, *maeA*, *frmR*, *fhuC*, *entF*, *fhuC*, and *entF* and *amn*) that show CS pattern. These proteins are underexplored; no FDA-approved molecules/inhibitors are available, and they can be considered indicators or regulators of the emergence of antimicrobial resistance and/or CS among bacterial pathogens. To the best of our knowledge, this is the first study in the domain to identify CS pairs for the last-resort antibiotic colistin in the treatment of colistin-resistant *K. pneumoniae* infections, which may also be explored in the future for their therapeutic and diagnostic utility (for staging AMR emergence in clinics).

## Data Availability

The datasets presented in this study can be found in online repositories. The names of the repository/repositories and accession number(s) can be found at: https://ibdc.dbt.gov.in/ipd/project-information.php?project_id=IPD5407.
