# Supplementary material for: Adaptive laboratory evolution and proteomic insight on collateral sensitivity for meropenem in colistin-resistant Klebsiella pneumoniae
Source: Front Microbiol. 2026 Oct 9;17:1960299. doi: 10.3389/fmicb.2026.1960299 (PMC13690759; doi:10.3389/fmicb.2026.1960299)
Supplement: Supplementary file 1 [file Data_Sheet_1.DOCX]

**Supplementary Data; contains different group comparison along with E-strip, AST based panel validation table of significant proteins list in colistin and meropenem with respect to ATCC and Clinical *Klebsiella pneumoniae***

**Supplementary Table 1**. All possible comparison groups

| ***K. pneumoniae* ATCC GA Colistin** | **Wild Type** | **0.25xMIC** | **0.5xMIC** | **1xMIC** |
| --- | --- | --- | --- | --- |
| Total Number of Identified proteins | 3126 | | | |
| Differentially expressed proteins (compared to control/WT) | NA | 36 | 258 | 247 |
| Upregulated |  | 23 | 194 | 147 |
| Down-regulated |  | 13 | 64 | 100 |

| ***K. pneumoniae* ATCC GA Meropenem** | **Wild Type** | **0.25xMIC** | **0.5xMIC** | **1xMIC** |
| --- | --- | --- | --- | --- |
| Total Number of Identified proteins | 3126 | | | |
| Differentially expressed proteins (compared to control/WT) | NA | 28 | 47 | 55 |
| Upregulated |  | 12 | 33 | 43 |
| Down-regulated |  | 16 | 14 | 12 |

| ***K. pneumoniae* ATCC GA Col1x-Meropenem** | **Wild Type** | **0.25xMIC** | **0.5xMIC** | **1xMIC** |
| --- | --- | --- | --- | --- |
| Total Number of Identified proteins | 3126 | | | |
| Differentially expressed proteins (compared to control/WT) | NA | 143 | 177 | 170 |
| Upregulated |  | 114 | 103 | 147 |
| Down-regulated |  | 29 | 74 | 23 |

| ***K. pneumoniae* Clinical GA Colistin** | **Wild Type** | **0.25xMIC** | **0.5xMIC** | **1xMIC** |
| --- | --- | --- | --- | --- |
| Total Number of Identified proteins | 3126 | | | |
| Differentially expressed proteins (compared to control/WT) | NA | 106 | 34 | 171 |
| Upregulated |  | 19 | 7 | 66 |
| Down-regulated |  | 87 | 27 | 105 |

| ***K. pneumoniae* Clinical GA Meropenem** | **Wild Type** | **0.25xMIC** | **0.5xMIC** | **1xMIC** |
| --- | --- | --- | --- | --- |
| Total Number of Identified proteins | 3126 | | | |
| Differentially expressed proteins (compared to control/WT) | NA | 591 | 532 | 565 |
| Upregulated |  | 338 | 296 | 328 |
| Down-regulated |  | 253 | 239 | 237 |

| ***K. pneumoniae* Clinical GA Col1x-Meropenem** | **Wild Type** | **0.25xMIC** | **0.5xMIC** | **1xMIC** |
| --- | --- | --- | --- | --- |
| Total Number of Identified proteins | 3126 | | | |
| Differentially expressed proteins (compared to control/WT) | NA | 360 | 216 | 308 |
| Upregulated |  | 291 | 151 | 221 |
| Down-regulated |  | 69 | 65 | 87 |

**Supplementary Figure 1**. *In-vitro* evaluation of minimum inhibitory concentration (MIC) by broth microdilution of different *Klebsiella pneumoniae* for colistin and meropenem**. (A)** *K. pneumoniae* (ATCC) exposed to colistin**; (B)** *K. pneumoniae* (ATCC) exposed to meropenem**; (C)** 1x colistin adapted *K. pneumoniae* ATCC exposed to meropenem; **(D)** *K. pneumoniae* (clinical) exposed to colistin**; (E)** *K. pneumoniae* (clinical) exposed to meropenem**; (F)** 1x colistin adapted *K. pneumoniae* clinical exposed to meropenem.

**Supplementary Figure 2**.- Validation of developed resistant panels**.** Growth based validation 0.25x, 0.5x, and 1x adapted *K. pneumoniae* **(A)** colistin adapted *K. pneumoniae* (ATCC); **(B)** meropenem adapted *K. pneumoniae* (ATCC); **(C)** meropenem adapted 1x colistin resistant *K. pneumoniae* (ATCC); **(D)** colistin adapted *K. pneumoniae* (Clinical); **(E)** meropenem adapted *K. pneumoniae* (Clinical); **(F)** meropenem adapted 1x colistin resistant *K. pneumoniae* (Clinical). Validation of developed resistant panels on Agar plate on E-strips**.** E-strips assay-based validation 0.25x, 0.5x, and 1x adapted *K. pneumoniae* **(G)** colistin adapted *K. pneumoniae* (ATCC); **(H)** meropenem adapted *K. pneumoniae* (ATCC); **(I)** meropenem adapted 1x colistin resistant *K. pneumoniae* (ATCC); **(J)** colistin adapted *K. pneumoniae* (Clinical); **(K)** meropenem adapted *K. pneumoniae* (Clinical); **(L)** meropenem adapted 1x colistin resistant *K. pneumoniae* (Clinical).

**Supplementary Table 2**. Pathogen Identification by MALDI-TOF MS rules out cross contamination.

| **Patient**  **ID** | **Patient Name** | **ID** | **Specimen**  **Type** | **Organism**  **Name** | **Confidence**  **Value** |
| --- | --- | --- | --- | --- | --- |
| 1 | KP_ATCC_WT | T-1-1 | Bacterial  Colony | *K. pneumoniae* | 99.99% |
| 2 | KP_ATCC GA_Col_0.25x | T-2-1 | Bacterial  Colony | *K. pneumoniae* | 99.99% |
| 3 | KP_ATCC GA_Col_0.5x | T-3-1 | Bacterial  Colony | *K. pneumoniae* | 99.99% |
| 4 | KP_ATCC GA_Col_1x | T-4-1 | Bacterial  Colony | *K. pneumoniae* | 99.99% |
| 5 | KP_ATCC GA_Col_1x- Mer0.25x | T-5-1 | Bacterial  Colony | *K. pneumoniae* | 99.99% |
| 6 | KP_ATCC GA_Col_1x- Mer0.5x | T-6-1 | Bacterial  Colony | *K. pneumoniae* | 99.99% |
| 7 | KP_ATCC GA_Col_1x- Mer 1x | T-7-1 | Bacterial  Colony | *K. pneumoniae* | 99.99% |
| 8 | KP_ATCC GA_Mer_0.25x | T-8-1 | Bacterial  Colony | *K. pneumoniae* | 99.99% |
| 9 | KP_ATCC GA_Mer_0.5x | T-9-1 | Bacterial  Colony | *K. pneumoniae* | 99.99% |
| 10 | KP_ATCC GA_Mer_1x | T-10-1 | Bacterial  Colony | *K. pneumoniae* | 99.99% |
| 11 | KP_Clinical_WT | T-11-1 | Bacterial  Colony | *K. pneumoniae* | 99.99% |
| 12 | KP_Clinical GA_Col_0.25x | T-12-1 | Bacterial  Colony | *K. pneumoniae* | 99.99% |
| 13 | KP_Clinical GA_Col_0.5x | T-13-1 | Bacterial  Colony | *K. pneumoniae* | 99.99% |
| 14 | KP_Clinical GA_Col_1x | T-14-1 | Bacterial  Colony | *K. pneumoniae* | 99.99% |
| 15 | KP_Clinical GA_Col_1x- Mer0.25x | T-15-1 | Bacterial  Colony | *K. pneumoniae* | 99.99% |
| 16 | KP_Clinical GA_Col_1x- Mer0.5x | T-16-1 | Bacterial  Colony | *K. pneumoniae* | 99.99% |
| 17 | KP_Clinical GA_Col_1x- Mer1x | T-17-1 | Bacterial  Colony | *K. pneumoniae* | 99.99% |
| 18 | KP_Clinical GA_Mer_0.25x | T-18-1 | Bacterial  Colony | *K. pneumoniae* | 99.99% |
| 19 | KP_Clinical GA_Mer_0.5x | T-19-1 | Bacterial  Colony | *K. pneumoniae* | 99.99% |
| 20 | KP_Clinical GA_Mer_1x | T-20-1 | Bacterial  Colony | *K. pneumoniae* | 99.99% |

**Supplementary Table 3**.- BioMerieux VITEK 2 AST Card No. N407 Antibiotics Details

|  | **BioMerieux VITEK 2 AST Card No. N407 Antibiotics Details** | | | |
| --- | --- | --- | --- | --- |
| **S.No.** | **Antibiotics** | **Code** | **Mode of action** | **Class** |
| **1** | **Ampicillin/Sulbactam** | SAM | Cell wall synthesis | Aminopenicillins |
| **2** | **Cefoxitin** | FOX | Cell wall synthesis (PBP) | Cephamycin |
| **3** | **Ceftizoxime** | CZX | Cell wall synthesis (PBP) | Third-Generation Cephalosporin |
| **4** | **Ceftazidime/Avibactam** | CZA | Cell wall synthesis (PBP) | Third-Generation Cephalosporin |
| **5** | **Ceftazidime/Tazobactam** | CT | Cell wall synthesis (PBP3) | Third-Generation Cephalosporin |
| **6** | **Cefepime** | FEP | Cell wall synthesis | Fourth-Generation Cephalosporin |
| **7** | **Meropenem** | MEM | Cell wall synthesis (PBP) | Carbapenems |
| **8** | **Netilmicin** | NET | Protein synthesis (30S) | Aminoglycoside Antibiotic |
| **9** | **Tobramycin** | TM | Protein synthesis (30S-50S) | Aminoglycoside Antibiotic |
| **10** | **Levofloxacin** | LEV | Bacterial DNA synthesis GyrB | Fluoroquinolones |
| **11** | **Doxycycline** | DO | Protein synthesis (30S) | Tetracycline  antibiotics |
| **12** | **Tetracycline** | TE | Protein synthesis (30S) | Tetracycline  antibiotics |
| **13** | **Chloramphenicol** | C | Protein synthesis (50S) | Amphenicol Antibiotic |
| **14** | **Colistin** | CS | Cell Membrane | Polymyxin Antibiotic |
| **15** | **Polymyxin B** | PB | Cell Membrane | Polymyxin Antibiotic |

**Supplementary** **Figure 3.-** Heatmap depicting antimicrobial susceptibility (AST) of resistant *K. pneumoniae* cells. Data is generated by an automated VITEK-2 system. Horizontal gaps among rows denote antibiotic categories based on mode of action. **(A)** *K. pneumoniae* (ATCC) exposed to colistin**; (B)** *K. pneumoniae* (ATCC) exposed to meropenem**; (C)** 1x colistin adapted *K. pneumoniae* ATCC exposed to meropenem; **(D)** *K. pneumoniae* (clinical) exposed to colistin**; (E)** *K. pneumoniae* (clinical) exposed to meropenem**; (F)** 1x colistin adapted *K. pneumoniae* clinical exposed to meropenem.

**Supplementary Figure 4.-** Volcano plots showing DEPs in GA colistin adapted *K. pneumoniae* ATCC: **(A)**-0.25x:36 DEPs, **(B)**-0.5x:258 DEPs, **(C)**-1x:247 DEPs. Volcano plots showing DEPs in GA Meropenem adapted *K. pneumoniae* ATCC: **(D)**-0.25x:28 DEPs, **(E)**-0.5x:47 DEPs, **(F)**-1x:55 DEPs. Volcano plots showing DEP’s in 1x colistin resistant *K. pneumoniae* ATCC volcano plots: **(G)**-0.25x:143 DEPs, **(H)**-0.5x:177 DEPs, **(I)**-1x:170 DEPs

 **Supplementary Figure 5.-** Volcano plots showing DEPs in GA colistin adapted *K. pneumoniae* Clinical volcano plots: **(A)**-0.25x:106 DEPs, **(B)**-0.5x:34 DEPs, **(C)**-1x:171 DEPs. Volcano plots showing DEPs in GA Meropenem adapted *K. pneumoniae* Clinical volcano plots: **(D)**-0.25x:591 DEPs, **(E)**-0.5x:532 DEPs, **(F)**-1x:565 DEPs. Volcano plots showing DEP’s in 1x colistin resistant *K. pneumoniae* Clinical volcano plots: **(G)**-0.25x:360 DEPs, **(H)**-0.5x:216 DEPs, **(I)**-1x:308 DEPs in adapted conditions.

**Supplementary Figure 6.1.-** Molecular Functions (GO) showing DEPs in GA colistin adapted *K. pneumoniae* ATCC: **(A)**-0.25x:36 DEPs, **(B)**-0.5x:258 DEPs, **(C)**-1x:247 DEPs. Molecular Functions (GO) showing DEPs in GA Meropenem adapted *K. pneumoniae* ATCC: **(D)**-0.25x:28 DEPs, **(E)**-0.5x:47 DEPs, **(F)**-1x:55 DEPs. Molecular Functions (GO) showing DEP’s in 1x colistin resistant *K. pneumoniae* ATCC: **(G)**-0.25x:143 DEPs, **(H)**-0.5x:177 DEPs, **(I)**-1x:170 DEPs.

**Supplementary Figure 6.2.-** Molecular Functions (GO) showing DEPs in GA colistin adapted *K. pneumoniae* Clinical: **(A)**-0.25x:106 DEPs, **(B)**-0.5x:34 DEPs, **(C)**-1x:171 DEPs. Molecular Functions (GO) showing DEPs in GA Meropenem adapted *K. pneumoniae* Clinical: **(D)**-0.25x:591 DEPs, **(E)**-0.5x:532 DEPs, **(F)**-1x:565 DEPs. Molecular Functions (GO) showing DEPs in 1x colistin resistant *K. pneumoniae* Clinical: **(G)**-0.25x:360 DEPs, **(H)**-0.5x:216 DEPs, **(I)**-1x:308 DEPs in adapted conditions.

**Supplementary Figure 7 A, B.-** Common DEPs proteins between ATCC and clinical *K. pneumoniae* strain showing either upward or downward expression. **A)** 23 (15 up, 8 down) and **B)** 5 (4 up, 1 down) proteins responsible for colistin and meropenem resistance respectively.

**Supplementary Table 7 (A, B, C and D)-** Red color-Down regulated, green color-Up-regulated

| **ATCC Col Vs Clinical Col** | **ATCC Mer Vs Clinical Mer** |
| --- | --- |
| **Total 23** | **Total 5** |
| Riboflavin synthase [OS=*Klebsiella pneumoniae*] | Small ribosomal subunit protein uS15 [OS=*Klebsiella pneumoniae* subsp. pneumoniae HS11286] |
| submitted name: Glutathionine S-transferase [OS=*Klebsiella pneumoniae*] | SH3b domain-containing protein [OS=*Klebsiella pneumoniae* subsp. pneumoniae MGH 78578] |
| glutathione-specific gamma-glutamylcyclotransferase [OS=*Klebsiella pneumoniae* subsp. ozaenae] | submitted name: KdgM family porin OmpK26 [OS=*Klebsiella pneumoniae* subsp. pneumoniae] |
| submitted name: Iron transporter [OS=*Klebsiella pneumoniae*] | Fatty acid oxidation complex subunit alpha [OS=*Klebsiella pneumoniae*] |
| submitted name: An aldehyde reductase [OS=*Klebsiella pneumoniae* subsp. pneumoniae MGH 78578] | **submitted name: ATP-binding component of a transport system [OS=*Klebsiella pneumoniae* subsp. pneumoniae HS11286]** |
| **submitted name: PhoP [OS=*Klebsiella pneumoniae*]** |  |
| **NAD-dependent malic enzyme [OS=*Klebsiella pneumoniae* subsp. pneumoniae]** |  |
| **submitted name: Histidine kinase [OS=*Klebsiella pneumoniae*]** |  |
| **submitted name: Outer membrane protein A [OS=*Klebsiella pneumoniae*]** |  |
| **Potassium-transporting ATPase ATP-binding subunit [OS=*Klebsiella pneumoniae*]** |  |
| **UDP-4-amino-4-deoxy-L-arabinose--oxoglutarate aminotransferase [OS=*Klebsiella pneumoniae* 30684/NJST258_2]** |  |
| **submitted name: Ferrichrome transport ATP-binding protein FhuC [OS=*Klebsiella pneumoniae*]** |  |
| **Probable 4-deoxy-4-formamido-L-arabinose-phosphoundecaprenol deformylase ArnD [OS=*Klebsiella pneumoniae*]** |  |

**Supplementary Table 7 C, D-** Red color-Down regulated, green color-Up-regulated

| **Reversal ATCC Mer Vs Clinical Mer** | **ATCC Col Vs ATCC Mer** |
| --- | --- |
| **Total-54** | **Total-20** |
| submitted name: DNA-binding transcriptional activator CadC [OS=*Klebsiella pneumoniae* subsp. ozaenae] | Fumarate hydratase class II [OS=*Klebsiella pneumoniae* 30684/NJST258_2] |
| C4-dicarboxylate transport protein [OS=*Klebsiella pneumoniae* 30684/NJST258_2] | SH3b domain-containing protein [OS=*Klebsiella pneumoniae* subsp. pneumoniae MGH 78578] |
| **submitted name: Enterobactin non-ribosomal peptide synthetase EntF [OS=*Klebsiella pneumoniae*]** | submitted name: Outer membrane protein A [OS=*Klebsiella pneumoniae*] |
| Deoxyguanosinetriphosphate triphosphohydrolase [OS=*Klebsiella pneumoniae*] | Fatty acid oxidation complex subunit alpha [OS=*Klebsiella pneumoniae*] |
| Phosphate-specific transport system accessory protein PhoU [OS=*Klebsiella pneumoniae* subsp. ozaenae] | submitted name: Autotransporter assembly complex protein TamB [OS=*Klebsiella pneumoniae*] |
| submitted name: AsmA protein [OS=*Klebsiella pneumoniae*] | Fumarate hydratase class II [OS=*Klebsiella pneumoniae* 30684/NJST258_2] |
| submitted name: Efflux (PET) family inner membrane protein YccS [OS=*Klebsiella pneumoniae*] | submitted name: An aldehyde reductase [OS=*Klebsiella pneumoniae* subsp. pneumoniae MGH 78578] |
| Soluble pyridine nucleotide transhydrogenase [OS=*Klebsiella pneumoniae* subsp. ozaenae] | submitted name: L-lysine N6-monooxygenase [OS=*Klebsiella pneumoniae* subsp. pneumoniae] |
| **Potassium-transporting ATPase ATP-binding subunit [OS=*Klebsiella pneumoniae*]** | Tetraacyldisaccharide 4'-kinase [OS=*Klebsiella pneumoniae* subsp. pneumoniae HS11286] |
| submitted name: Phosphoglycerol transferase I [OS=*Klebsiella pneumoniae*] | Coenzyme A biosynthesis bifunctional protein CoaBC [OS=*Klebsiella pneumoniae* 30684/NJST258_2] |
| Phosphate regulon sensor protein PhoR [OS=*Klebsiella pneumoniae* subsp. pneumoniae] | Soluble pyridine nucleotide transhydrogenase [OS=*Klebsiella pneumoniae* subsp. ozaenae] |
| **submitted name: Modulating protein YMOA [OS=*Klebsiella pneumoniae* 30684/NJST258_2]** | Bifunctional aspartokinase/homoserine dehydrogenase [OS=*Klebsiella pneumoniae*] |
| Tetratricopeptide repeat protein [OS=*Klebsiella pneumoniae*] | submitted name: 2-dehydro-3-deoxygluconokinase [OS=*Klebsiella pneumoniae* 30684/NJST258_2] |
| submitted name: Transcriptional regulator RutR [OS=*Klebsiella pneumoniae* subsp. pneumoniae] | 23S rRNA (uracil(747)-C(5))-methyltransferase RlmC [OS=*Klebsiella pneumoniae* subsp. pneumoniae] |
| submitted name: Dihydrodipicolinate synthase family protein [OS=*Klebsiella pneumoniae*] | Fatty acid oxidation complex subunit alpha [OS=*Klebsiella pneumoniae*] |
| **NAD-dependent malic enzyme [OS=*Klebsiella pneumoniae* subsp. pneumoniae]** | submitted name: Putative HTH-type transcriptional regulator abgR [OS=*Klebsiella pneumoniae* 342] |
| Glycosyltransferase [OS=*Klebsiella pneumoniae*] | Ribose-5-phosphate isomerase C-terminal domain-containing protein [OS=*Klebsiella pneumoniae*] |
| submitted name: Ornithine decarboxylase [OS=*Klebsiella pneumoniae*] | submitted name: Putative 2-deoxy-D-gluconate 3-dehydrogenase [OS=*Klebsiella pneumoniae*] |
| **submitted name: PhoP [OS=*Klebsiella pneumoniae*]** | submitted name: Nucleotide di-P-sugar epimerase or dehydratase [OS=*Klebsiella pneumoniae*] |
| Undecaprenyl phosphate-alpha-4-amino-4-deoxy-L-arabinose arabinosyl transferase [OS=*Klebsiella pneumoniae* 30684/NJST258_2] | submitted name: Translocation protein TolB [OS=*Klebsiella pneumoniae* subsp. pneumoniae HS11286] |
| Lipoprotein [OS=*Klebsiella pneumoniae* 30684/NJST258_2] |  |
| submitted name: Sulfate and thiosulfate binding protein CysP [OS=*Klebsiella pneumoniae*] |  |
| Sulfate adenylyltransferase subunit 2 [OS=*Klebsiella pneumoniae*] |  |
| Sulfate adenylyltransferase subunit 1 [OS=*Klebsiella pneumoniae*] |  |
| Propionate 3-nitronate monooxygenase [OS=*Klebsiella pneumoniae* subsp. pneumoniae HS11286] |  |
| submitted name: Outer membrane lipoprotein SlyB [OS=*Klebsiella pneumoniae* subsp. pneumoniae] |  |
| AMP nucleosidase [OS=*Klebsiella pneumoniae* subsp. pneumoniae HS11286] |  |
| **NAD-dependent malic enzyme [OS=*Klebsiella pneumoniae* subsp. pneumoniae]** |  |
| submitted name: Ornithine decarboxylase [OS=*Klebsiella pneumoniae*] |  |
| Glycosyltransferase [OS=*Klebsiella pneumoniae*] |  |
| submitted name: Dihydrodipicolinate synthase family protein [OS=*Klebsiella pneumoniae*] |  |
| submitted name: 2-dehydro-3-deoxy-D-gluconate 5-dehydrogenase KduD [OS=*Klebsiella pneumoniae*] |  |
| Lipoprotein [OS=*Klebsiella pneumoniae* 30684/NJST258_2] |  |
| **submitted name: PhoP [OS=*Klebsiella pneumoniae*]** |  |
| submitted name: Transcriptional regulatory protein basR/pmrA [OS=*Klebsiella pneumoniae* 30684/NJST258_2] |  |
| submitted name: Translocation protein TolB [OS=*Klebsiella pneumoniae* subsp. pneumoniae HS11286] |  |
| **submitted name: Ferrichrome transport ATP-binding protein FhuC [OS=*Klebsiella pneumoniae*]** |  |
| **UDP-4-amino-4-deoxy-L-arabinose--oxoglutarate aminotransferase [OS=*Klebsiella pneumoniae* 30684/NJST258_2]** |  |
| Undecaprenyl-phosphate 4-deoxy-4-formamido-L-arabinose transferase [OS=*Klebsiella pneumoniae*] |  |
| Enoyl reductase (ER) domain-containing protein [OS=*Klebsiella pneumoniae* 30684/NJST258_2] |  |
| submitted name: Ornithine decarboxylase [OS=*Klebsiella pneumoniae*] |  |
| submitted name: Deoxyribodipyrimidine photolyase [OS=*Klebsiella pneumoniae* subsp. pneumoniae HS11286] |  |
| **AMP nucleosidase [OS=*Klebsiella pneumoniae* subsp. pneumoniae HS11286]** |  |
| **Transcriptional repressor FrmR [OS=*Klebsiella pneumoniae*]** |  |
| submitted name: ElaB protein [OS=*Klebsiella pneumoniae* 30684/NJST258_2] |  |
| submitted name: Deoxyguanosinetriphosphate triphosphohydrolase [OS=*Klebsiella pneumoniae* subsp. pneumoniae] |  |
| Amino-acid acetyltransferase [OS=*Klebsiella pneumoniae*] |  |
| Ribose-5-phosphate isomerase C-terminal domain-containing protein [OS=*Klebsiella pneumoniae*] |  |
| Altronate oxidoreductase [OS=*Klebsiella pneumoniae*] |  |
| Phosphatidylglycerophosphatase C [OS=*Klebsiella pneumoniae*] |  |
|  |  |

**Supplementary Table 7 E-** Red color-Down regulated, green color-Up-regulated

**Clinical Col Vs Clinical Mer**

Total- 23

submitted name: Aminoglycoside (6')-acetyltransferase [OS=*Klebsiella pneumoniae*]

submitted name: PTS N N'-diacetylchitobiose transporter subunit IIA [OS=*Klebsiella pneumoniae*]

submitted name: Amidohydrolase [OS=*Klebsiella pneumoniae*]

submitted name: Glucosamine-6-phosphate isomerase [OS=*Klebsiella pneumoniae* 30684/NJST258_2]

Acyl-coenzyme A dehydrogenase [OS=*Klebsiella pneumoniae*]

submitted name: Aminoglycoside (6')-acetyltransferase [OS=*Klebsiella pneumoniae*]

Anaerobic ribonucleoside-triphosphate reductase-activating protein [OS=*Klebsiella pneumoniae*]

Sirohydrochlorin cobaltochelatase [OS=*Klebsiella pneumoniae*]

Sucrose-6-phosphate hydrolase [OS=*Klebsiella pneumoniae*]

submitted name: Glucosamine-fructose-6-phosphate aminotransferase [OS=*Klebsiella pneumoniae* subsp. pneumoniae HS11286]

Nicotinate-nucleotide--dimethylbenzimidazole phosphoribosyltransferase [OS=*Klebsiella pneumoniae*]

Citrate-sodium symporter [OS=*Klebsiella pneumoniae*]

submitted name: Oxidoreductase FeS-binding subunit [OS=*Klebsiella pneumoniae*]

submitted name: High-affinity leucine-specific transport system [OS=*Klebsiella pneumoniae*]

Cold shock-like protein CspD [OS=*Klebsiella pneumoniae* 30684/NJST258_2]

submitted name: Integral membrane protein YfiB [OS=*Klebsiella pneumoniae* subsp. pneumoniae]

Siroheme synthase [OS=*Klebsiella pneumoniae*]

glutathione-specific gamma-glutamylcyclotransferase [OS=*Klebsiella pneumoniae* subsp. ozaenae]

peptidase Do [OS=*Klebsiella pneumoniae*]

Maltose/maltodextrin transport system permease protein [OS=*Klebsiella pneumoniae* 30684/NJST258_2]

submitted name: Enterobactin non-ribosomal peptide synthetase EntF [OS=*Klebsiella pneumoniae*]

SH3b domain-containing protein [OS=*Klebsiella pneumoniae* subsp. pneumoniae MGH 78578]

submitted name: Enterobactin esterase [OS=*Klebsiella pneumoniae*]

**Supplementary Figure 7 C.-** Common DEPs proteins between ATCC and clinical strains showing either upward or downward expression Total 54 DEP’s (6 up, 48 down) responsible for 1x colistin resistant *K. pneumoniae* adaptation to meropenem.

**Supplementary Figure 7 D.-** DEPs responsible for meropenem (at 0.25x, 0.5x, and 1x) and colistin (at 0.25x, 0.5x, and 1x) adaptation in *K. pneumoniae* (ATCC strain) showing either upward or downward expression. Total of 20 (17 up, 3 down).

**Supplementary Figure 7 E.-** DEPs responsible for meropenem (at 0.25x, 0.5x, and 1x) and colistin (at 0.25x, 0.5x, and 1x) adaptation *K. pneumoniae* (clinical strain) showing either upward or downward expression. Total of 23 (6 up, 17 down).

**Supplementary Figure 8.-** Expression of SH3b domain containing protein at 0.25x, 0.5x, and 1x adaptation in both ATCC and clinical strain studies (against both antibiotics colistin and meropenem).
